# Biofinder detects biological remains in Green River fish fossils from Eocene epoch at video speed

**DOI:** 10.1038/s41598-022-14410-8

**Published:** 2022-06-17

**Authors:** Anupam K. Misra, Sonia J. Rowley, Jie Zhou, Tayro E. Acosta-Maeda, Luis Dasilveira, Gregory Ravizza, Kenta Ohtaki, Tina M. Weatherby, A. Zachary Trimble, Patrick Boll, John N. Porter, Christopher P. McKay

**Affiliations:** 1 Hawai‘i Institute of Geophysics and Planetology, University of Hawai‘i at Mānoa, Honolulu, HI 96822 USA; 2 Department of Earth Sciences, University of Hawai‘i at Mānoa, Honolulu, HI 96822 USA; 3 Department of Electrical Engineering, University of Hawai‘i at Mānoa, Honolulu, HI 96822 USA; 4 Pacific Biosciences Research Center, University of Hawai‘i at Mānoa, Honolulu, HI 96822 USA; 5 Department of Mechanical Engineering, University of Hawai‘i at Mānoa, Honolulu, HI 96822 USA; 6 NASA Ames Research Center, Moffett Field, Mountain View, CA 94035 USA

**Keywords:** Biological techniques, Planetary science, Optics and photonics

## Abstract

The “Search for life”, which may be extinct or extant on other planetary bodies is one of the major goals of NASA planetary exploration missions. Finding such evidence of biological residue in a vast planetary landscape is an enormous challenge. We have developed a highly sensitive instrument, the “Compact Color Biofinder”, which can locate minute amounts of biological material in a large area at video speed from a standoff distance. Here we demonstrate the efficacy of the Biofinder to detect fossils that still possess strong bio-fluorescence signals from a collection of samples. Fluorescence images taken by the Biofinder instrument show that all *Knightia* spp. fish fossils analysed from the Green River formation (Eocene, 56.0–33.9 Mya) still contain considerable amounts of biological residues. The biofluorescence images support the fact that organic matter has been well preserved in the Green River formation, and thus, not diagenetically replaced (replaced by minerals) over such a significant timescale. We further corroborated results from the Biofinder fluorescence imagery through Raman and attenuated total reflection Fourier-transform infrared (ATR-FTIR) spectroscopies, scanning electron microscopy, energy dispersive X-ray spectroscopy (SEM–EDS), and fluorescence lifetime imaging microscopy (FLIM). Our findings confirm once more that biological residues can survive millions of years, and that using biofluorescence imaging effectively detects these trace residues in real time. We anticipate that fluorescence imaging will be critical in future NASA missions to detect organics and the existence of life on other planetary bodies.

## Introduction

The quest for finding evidence of present or past life on other planetary bodies is an important goal of several international space agencies, which may provide an answer to the questions “are we alone in the universe” and “was life ever present on other planets”?^1^ Since 2004, from the successful landings of NASA’s Mars Exploration Rovers, Opportunity (2004)^2–5^, Spirit (2004)^6^, Curiosity (2012)^7^, and Perseverance (2021)^8^ we have seen thousands of images from Mars of the Martian landscape. These images reveal mostly soil and rocks with no obvious features that suggest the evidence of life. The task of finding evidence of life has proven to be challenging. This is because possible life forms could most likely be small and/or extinct. Thus, research efforts have focused on the search for biosignatures: substances or phenomena that provide evidence of past or present life. Organic chemicals formed by biological processes or biogenic minerals are potential biosignatures: proteins, lipids, and the residues of fossils. The detection of such biomarkers would constitute groundbreaking evidence for life outside of planet Earth. This would be a breakthrough discovery and the ultimate goal of the search for life exploration programs. Considering the difficulty and yet critical importance of the task, it follows that there is a tremendous need for a remote sensing instrument that can detect minute biomarkers and possible life precursors in a wide area with a fast detection time. Here, we provide a solution to this problem with the development of the “Compact Color Biofinder”, which detects trace quantities of organic matter in a large area at video speed from a standoff distance of a few centimeters to 5 meters^1,9^. Similarly, other groups have developed fluorescence based instruments such as WALI—Wide Angle Laser Imaging^10^, and OrganiCam^11^.

Our team has been working since 2012 on the development of the Standoff Biofinder^12,13^ with the intent of the rapid detection and location of biological materials in a wide geological context for planetary exploration. Most biological materials e.g., amino acids, fossils, clays, sedimentary rocks, plants, microbes, bio-residues, proteins, lipids, etc. have strong fluorescence signals, which also have a very short lifetime of less than 20 ns^14–16^. Polycyclic aromatic hydrocarbon (PAHs), abiotic organics (amino acids, plastic etc.) also have short fluorescence lifetime and show up in the Biofinder fluorescence images. The Biofinder instrument uses this short-lived fluorescence property of organic materials to detect and locate them in a large area at video speed. Since its creation, the instrument has been proven to distinguish between mineral phosphorescence and organic fluorescence from standoff distances in daylight conditions with short measurement times (1 µs), and to differentiate between different organic materials by taking colour images^1,9,17^. These capabilities could help an exploration rover to identify objects of interest for the ‘search for life’ beyond planet Earth and then guide other characterization techniques, such as Raman or Laser-Induced Breakdown Spectroscopy (LIBS), to determine the molecular and elemental composition of the selected targets.

## Results and discussion

In this work, we have used the Biofinder instrument from a distance of 50 cm to screen through a collection of thirty-five *Knightia* spp. of fish^18^ fossil specimens from the Green River formation^19,20^, Colorado, Wyoming, and Utah, USA (Eocene, 56.0–33.9 Mya) in their original rock matrix to look for signs of biological remains. Fossils are preserved remains from biological entities from past geological ages and the Green River formation has been known for its excellent preservation of fossils. The Biofinder instrument showed that all of the *Knightia* fish fossils still contain a significant quantity of bio-fluorescence. The goal of this work was to perform data validation of the Biofinder results and confirm the presence of organic materials in fossil specimens using gold standard techniques, namely Raman^21,22^ and attenuated total reflection Fourier-transform infrared (ATR-FTIR)^23,24^, which provide molecular evidence through vibrational modes; scanning electron microscopy and energy dispersive X-ray spectroscopy (SEM–EDS), which provides elemental composition at microscopic scale; and fluorescence lifetime imaging microscopy (FLIM)^25^, which is a standard technique in biological research based on the short fluorescence lifetime of organic material. The successful data validation presented here demonstrates that biological residues can survive millions of years. These results corroborate with the recent findings of biomarkers in fossils^26–43^. Fluorescence microscopes^44^ and examination under UV illumination^45,46^ have been used in the past to investigate fossils. These methods are limited in their capability because of difficulty in performing in-situ detection during daylight and inability to separate out interferences from mineral phosphorescence. The Standoff Biofinder provides a rapid, standoff, in-situ method of biomarker detection, which is unlike previous detection methods and instrumentation. Thus, this work provides clear evidence to support the usage of the Standoff Biofinder in many applications requiring accurate and fast detection of biological life; for example, detecting extinct or extant life on other planetary bodies in future NASA missions.

The Biofinder is a portable instrument that is operated using a 24 V battery and laptop (see extended data Fig. 1). The Biofinder instrument uses a compact solid state, conductively cooled Nd:YAG nano-second pulsed laser providing two simultaneous wavelengths, 355 and 532 nm, for fluorescence excitation. The system runs at a video speed of 20 frames/second, which is synchronized with the laser repetition rate of 20 pulses/s. For organic and biological fluorescence imaging the detector is gated for its shortest exposure time of 1 µs and records the short-lived fluorescence signals from organics and biological materials. Short detection time is helpful in blocking off the long-lived mineral phosphorescence and background signal from the day/room light^1,13^.

**Figure 1:**
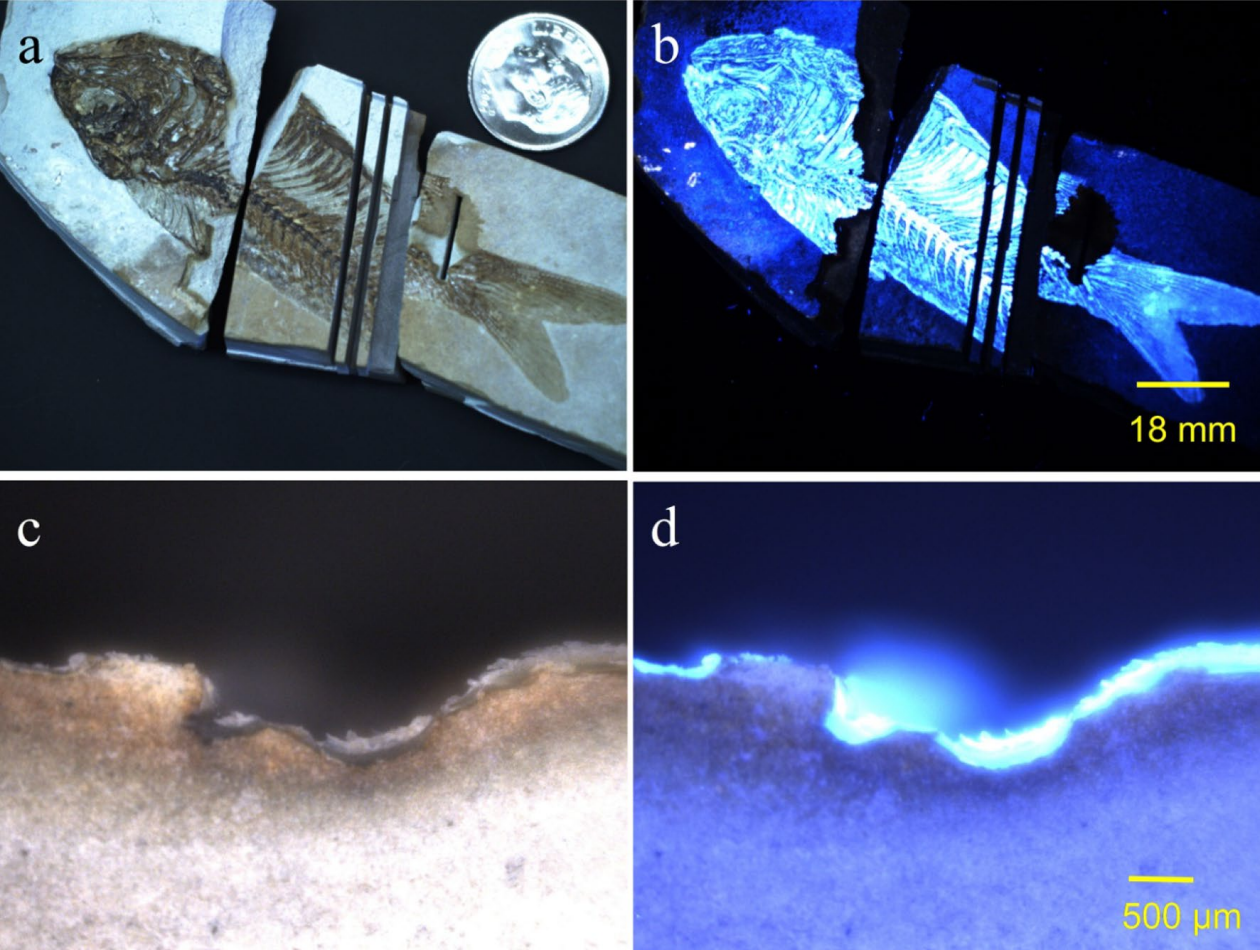
Biofinder detection of biological resides in fish fossil. (**a**) White light image of a Green River formation fish fossil, *Knightia* sp., from a distance of 50 cm using the Biofinder without laser excitation. (**b**) Fluorescence image of the fish fossil obtained by the Biofinder using a single laser pulse excitation, 1 µs detection time, and 3.6% gain on the CMOS detector. (**c**) Close-up white light image of the fish fossil cross-section using a 10× objective with 54 mm working distance showing the fish remains and rock matrix. (**d**) Fluorescence image with a single laser pulse excitation showing strong bio-fluorescence from the fish remains.

The Biofinder instrument also records regular white light colour images using day/room light as the source of illumination, and not the laser (Fig. 1a). Comparative images of the same *Knightia* sp. fossil using laser excitation (Fig. 1b) shows a very clear and strong bio-fluorescence signal in the blue spectra region when imaged by the Biofinder in the fluorescence imaging mode with a detector gain of 3.6%. To further test the detection capability of the Biofinder the camera lens was changed to a long working distance microscope objective, thus turning the instrument into a standoff fluorescence microscope. The same fossil was cut into several pieces to be imaged in cross-section (Fig. 1c). At the microscopic scale, fluorescence images (Fig. 1d) demonstrated the clear presence of organic material in the fossil by the characteristic fluorescence of organic matter detected using a 10× objective at a working distance of 54 mm. The brown color material has been known to paleontologists to be organic matter formed from residues of fish bones along with soft tissues^20^ and hence, we can say that the organic fluorescence comes from biological origin. The Fig. 1d shows that biological fluorescence is very strong in the translucent layer, which is present on top of the brown sediment layer.

**Figure 2:**
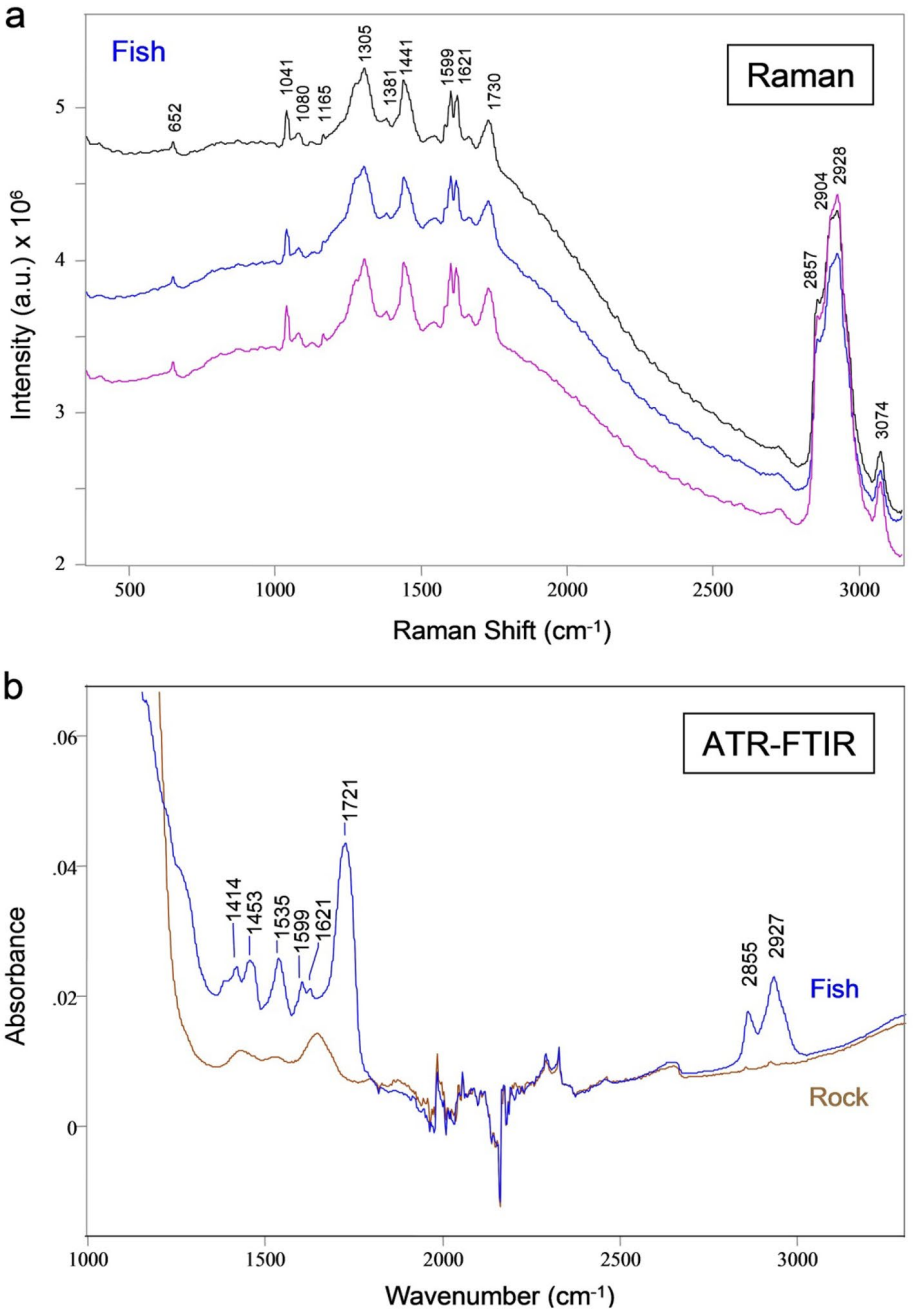
Confirmation of organics in fish fossil using Raman and ATR-FTIR spectroscopy. (**a**) Raman spectra of the fish fossil remains showing Raman bands in the 2800–3100 cm^−1^ indicating the presence of intact C–H bonding in a fossil that is millions of years old. The Raman peaks in the 1100–1750 cm^−1^ region have typical spectral features, which indicate the presence of biological material. (**b**) ATR-FTIR spectra of fish fossil confirming the presence of organics in the same vibrational spectral region as the Raman spectra.

To investigate the molecular composition of the fossil remains two vibrational spectroscopy techniques were used; Raman and Attenuated total reflection Fourier-transform infrared (ATR-FTIR). Vibrational modes are very specific to each molecule; therefore, vibrational spectroscopy is considered a fingerprint technology for molecular identification with very high confidence level in detection. The Raman spectra of the fish fossil with an excitation of 785 nm (Fig. 2a) and the Raman peaks observed over the luminescence background show the presence of carbonate and a mixture of biological residues with C–C, C = C, C=O, C–H bonds. The vibrational mode assignment (Table 1) of the Raman peaks observed in the fish fossil are based on Raman literature^47^. Raman peaks with good signal to noise ratio in the 2800–3100 cm^−1^ region confirms the presence of CH bonding in the fossil sample. These results further validate the fact that biological residues in the Green River fossils have been well preserved over millions of years. Similarly, ATR-FTIR spectra^48^ (Fig. 2b) shows clear biological features in the *Knightia* sp. fossil in comparison to the rock matrix.

**Table 1:** Raman and ATR-FTIR vibrational modes assignment for fish fossil.

Raman peak position (cm^−1^)	Assignment	ATR-FTIR peak position (cm^−1^)	Assignment
652	C–C twisting		
1041	C–C stretching		
1080	Carbonate sym stretch		
1165	C–H in plane bending		
1275 (sh)	C=CH deformation		
1305	CH_2_ twist/bending		
1381	CH_3_ deformation lipid	1414	C-H deformation
1441	CH_2_ bending lipid	1453	CH_3_ bending lipid
1581	C=C bending	1535	C=C
1599	C=O stretching	1599	C=O stretching
1621	C=C stretching	1621	C=C stretching
1730	C=O stretching lipid	1721	C=O stretching
2857	CH_2_ sym stretch lipid	2855	CH_2_ stretch lipid
2904	CH_2_ asy stretch		
2928	CH_3_ sym stretch	2927	CH stretch
3074	CH asy stretch		

sh: Shoulder, sym: symmetric, asy: asymmetric.

**Figure 3:**
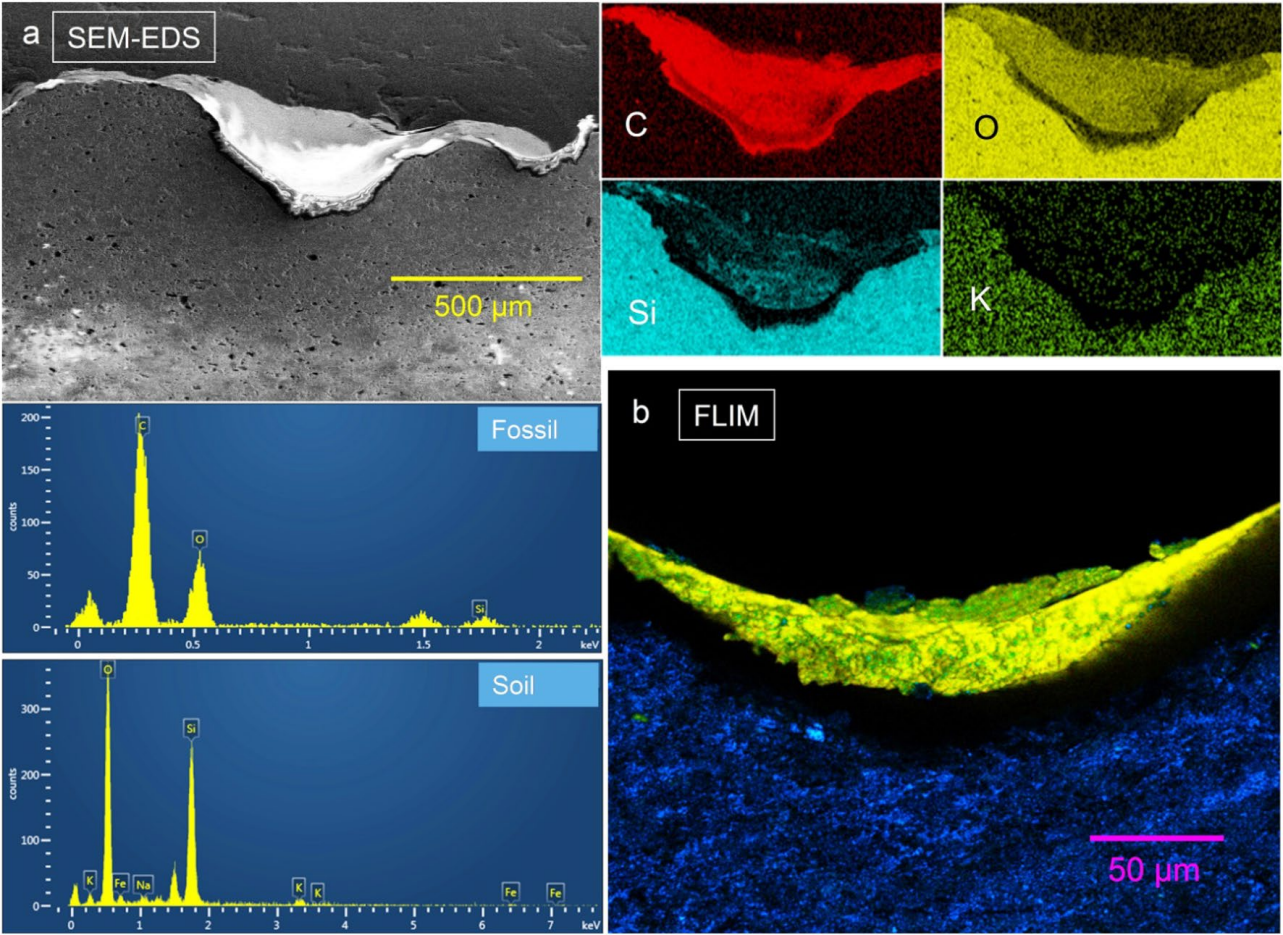
Confirmation of carbon and short-lived biofluorescence in fish fossil. (**a**) SEM–EDS analysis of the fish fossil cross-section showing that the fossil contains considerable quantities of carbon in comparison to the rock matrix. The rock matrix is rich in silica and has more oxygen than the fish. (**b**) FLIM image of the fossil cross-section showing strong bio-fluorescence in the fish (shown as false-coloured green-yellow region) with a lifetime of 2.7 ns.

The elemental composition of the fish fossil using SEM–EDS, clearly reveal that a very high level of carbon is present in comparison with the adjacent rock matrix (Fig. 3a). The EDS elemental analysis showed carbon at 65.7 wt%, oxygen 32.0 wt%, and silicon 2.3 wt% in the fish area and oxygen 58.7 wt%, silicon 36.7 wt%, potassium 3.5 wt%, sodium 1.1 wt% in the rock. The FLIM instrument was further used to determine the fluorescence lifetime of the biological materials in the sample, which showed a fluorescence lifetime of 2.7 ns in the fish area (shown as false colour green–yellow region) and 0.1 ns in the rock (shown as false colour blue region; Fig. 3b).

Data shown in Figs. 2 and 3 confirm regions of high organic remains in a fossil sample from the Eocene Epoch (56.0–33.9 Mya) that were detected by the Biofinder as shown in Fig. 1. Raman spectroscopy is very specific and provides molecular detection at almost 100% confidence level for most inorganic and organic chemicals because no two chemicals have the same Raman spectra. Because biological materials contain various types of molecules, Raman spectra of biological materials, however, give broad features that make it difficult to identify the exact chemical composition. Based on the Raman and EDS data, it appears that biological remains in the fossil fish area are most likely a mixture of biological materials with lipids as the primary component. A strong smell corresponding to crude oil was detected whilst making the thin sections of the sample using a diamond saw which is expected from an oil shale. This indicated the presence of lipids in the fossil region. Lipids are an essential part of living cells for energy storage. Moreover, lipids have been suggested as a universal biomarker for the detection of life due to their long-time chemical stability over millions of years^49–53^.

Extended data Fig. 2 shows Biofinder’s capability to detect the region of organic matter in 3.43 Gya old stromatolite from Strelley Pool, Western Australia. The sample was washed with water and dried to clean the outer surfaces. In addition, the sample was broken in the middle to reveal the interior structure (Fig. 2b) which was not cleaned with water. In some cases, fluorescence imaging can help to distinguish between abiotic organic residues from biological materials based on morphology. In Extended data Fig. 3, the detection of legs in a 95 Mya old shrimp fossil (from Sannine formation, Hjoula, Lebanon) in the fluorescence image is helpful in claiming the organic matter to be biotic.

## Conclusion

In conclusion, we demonstrate the capability of a highly sensitive instrument, the Biofinder, to detect the biological remains of fossils that are millions of years old at video speed using fast fluorescence signals. The findings from the Biofinder were confirmed using gold standard techniques, i.e., Raman, ATR-FTIR, SEM–EDS, and FLIM. This work demonstrates that the organic remains preserved in fossilized material for millions of years can be detected from a standoff distance with video speed in the presence of daylight and mineral phosphorescence. Standoff bio-fluorescence imaging can, thus, provide a significant capability to the future NASA’s “search for life” missions to find even minute evidence of life in a large geological landscape through rapid and effective scanning.

## Method summary

### Sample

Thirty-five *Knightia* spp. fish fossils from the Green River formation, Colorado, Wyoming, and Utah, USA (Eocene, 56.0–33.9 Mya) in their original rock matrix were purchased from various fossil vendors. Samples were imaged using the Biofinder without prior cleaning. For further downstream analyses samples were cut into thin sections using a slow speed diamond saw and water as the cooling media. Thin sections were dried and analyzed with Raman and ATR-FTIR without further processing. For SEM–EDS, samples were sonicated in ethanol for 60 s to remove surface contamination and dried.

### Compact color Biofinder (CoCoBi)

Images shown in Fig. 1a and b were recorded with the Biofinder instrument shown in Extended Data Fig. 1 from a distance of 50 cm. The working principle and instrumentation of the Biofinder instrument is described in detail in Misra et al.^1^. In brief, the Biofinder instrument uses two laser wavelengths (355 and 532 nm) and simultaneously illuminates a wide area that is under investigation to excite luminescence signals from the targets. A compact gated color CMOS detector is used to image only the short-lived fluorescence signals from organic materials and to prevent daylight and long-lived mineral phosphorescence background interference. The laser beam is expanded using a diffuser and a lens to uniformly illuminate a wide area of interest. For comparison, Fig. 1a was recorded with white light illumination without the laser in operation and using room lights as source. The corresponding fluorescence image (Fig. 1b) was recorded with a single laser pulse excitation and gated integration time of 1 µs with the room lights left on. The solid state conductively cooled Nd:YAG laser fires simultaneous 355, 532 and 1064 nm nano-second pulses (6 ns pulse width) at a repetition rate of 20 pulses/s. The 1064 nm beam was blocked using a short pass filter mounted in front of the laser optics. The CMOS camera was synchronized with the laser excitation using the trigger pulses provided by the laser. The short lifetime of the bio-fluorescence signal was verified by delaying the camera by another 1 µs and taking an image with 1 µs exposure, which showed complete darkness.

For close-up images shown in Fig. 1c and d, the 35 mm Fujinon camera lens of the Biofinder was replaced with a long working distance 10× microscope objective lens with a working distance of 54 mm. Both laser power and camera gain were reduced to obtain the fluorescence image shown in Fig. 1d.

### Raman spectra

The Raman spectra of the fish fossil shown in Fig. 2a were recorded with a commercial micro-Raman RXN system from Kaiser Optical Systems, Inc. (KOSI), utilizing a 785-nm laser excitation. A 50-micron slit width was used for measuring the micro-Raman spectra of samples mounted on an aluminum substrate. Raman spectra were recorded at several areas of the fossil fish bones using 50× objective, 100 mW of laser power, and 300 s integration time. Three representative spectra are presented here without the baseline removal showing luminescence background. The spectra have been shifted vertically for display purposes.

### Attenuated total reflection Fourier-transform infrared (ATR-FTIR)

ATR-FTIR spectra were recorded with a ThermoFisher Scientific Nicolet iS50 FT-IR spectrometer with an integrated Attenuated Total Reflection (ATR) module using a diamond crystal. Small grains of samples were compressed on the diamond crystal and spectra were measured with an average of 128 scans at room temperature. Before each sample measurement, the diamond crystal was cleaned with methanol and a background was obtained using the open optical path.

### Scanning electron microscopy and energy dispersive x-ray spectroscopy (SEM–EDS)

The cross-section areas of the fish fossil samples were surveyed with secondary electron imaging system without any conductive coating. The elemental distribution was mapped using energy dispersive x-ray spectroscopy at 15 kV with 0.2 nA using the FEI Helios 660 dual-beam focused ion beam instrument (FIB-SEM) equipped with an Oxford Instrument X-MaxN 80 silicon drift detector system in the Advanced Electron Microscopy Center at the University of Hawai’i, USA. The chemical compositions were also extracted from the elemental maps collected using Aztec software (Oxford Instruments, UK).

### Fluorescence lifetime imaging microscopy (FLIM)

FLIM imaging and analysis were performed on a Leica TCS SP8 X with Leica FALCON FLIM module (Leica Microsystems, Germany). Observations were made with a HC PL FLUOTAR 10x/0.30 dry lens. A pulsed white light laser operating at 80 MHz frequency was used for the fluorescence lifetime measurement.

## Supplementary Information

Supplementary Information.

## Data Availability

All data generated or analyzed during this study are included in this published article [and its supplementary information file].
