# Supplementary material for: Biofinder detects biological remains in Green River fish fossils from Eocene epoch at video speed
Source: Sci Rep. 2022 Jun 17;12:10164. doi: 10.1038/s41598-022-14410-8 (PMC9205911; doi:10.1038/s41598-022-14410-8)
Supplement: Supplementary file 1 — Supplementary Information. [file 41598_2022_14410_MOESM1_ESM.pdf]

Supplementary information:

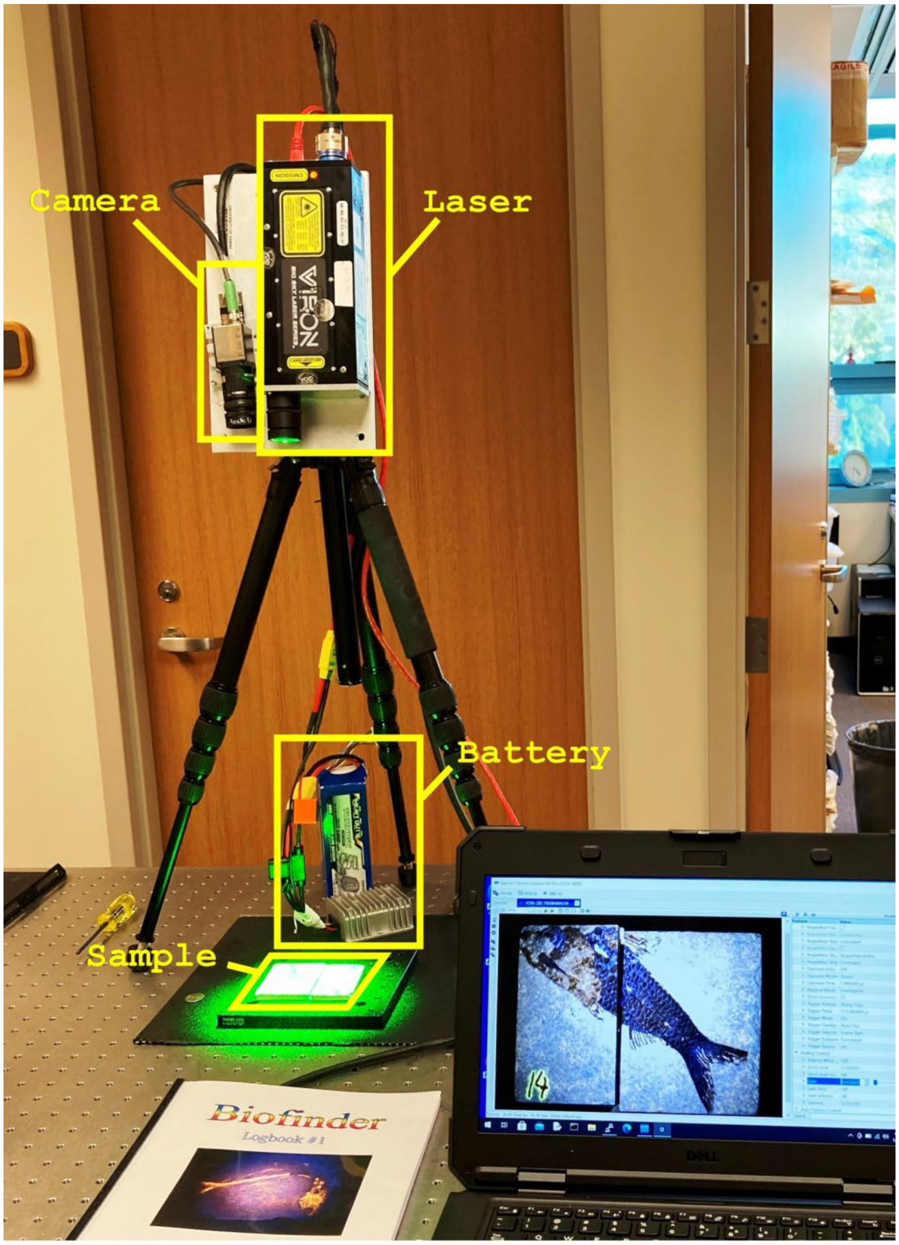

Extended Data Figure 1 | Portable standoff compact color Biofinder operating with 24 V battery and laptop. The Biofinder was used to screen fossil samples from 50 cm distance for evidence of bio-fluorescence in real time. The image shows the experimental setup and detection of bio-fluorescence from a distance of 50 cm with room lights on with 1 µs integration time in one of the fish fossil which is cut in half. The laptop image shows strong fluorescence signal in both the fish fossil and the rock matrix with detector gain of 10%. The instrument runs at video speed of 20 frames/second synchronized with the laser repetition rate of 20 pulses/second.

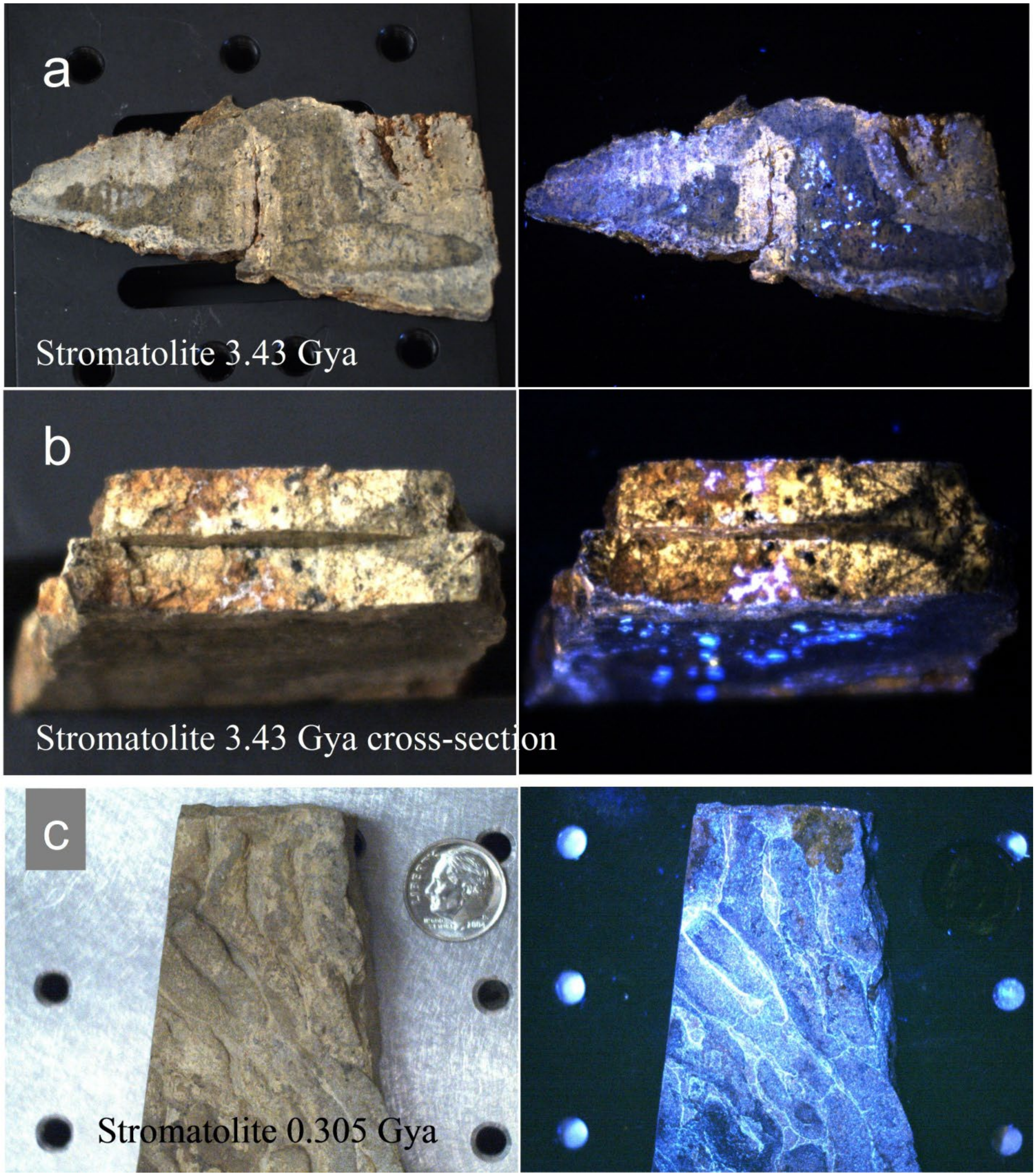

Extended Data Figure 2 | **Biofinder's images showing presence of organics in stromatolite**. **a,** 3.43 Gya old stromatolite from Strelley Pool , Western Australia which was broken in the middle using a chisel and hammer to reveal freshly exposed cross-section view shown in **b**. **c,** 305 Mya old stromatolite from Haskall County, Oklahoma (bottom images). White light images (left), Fluorescence images (right).

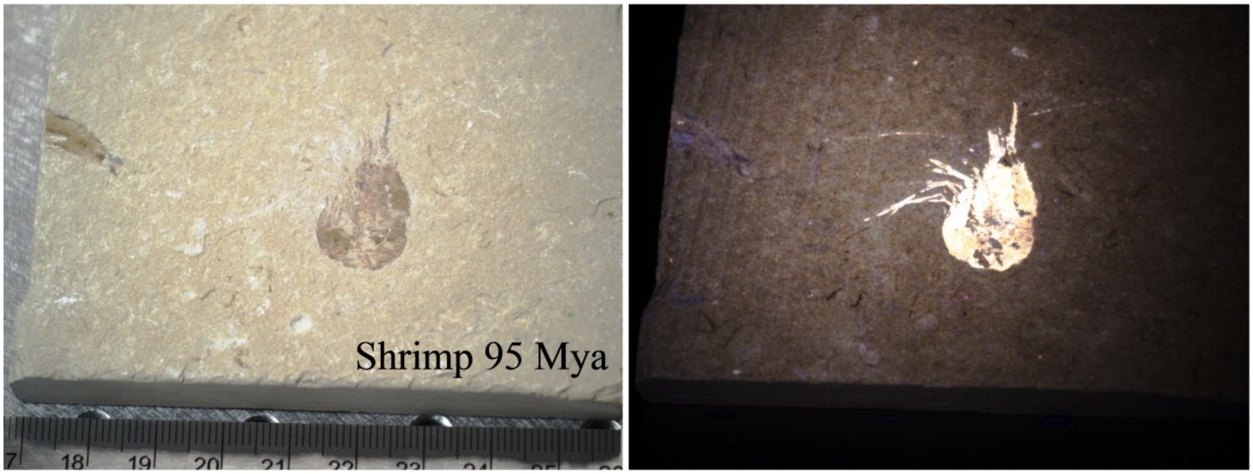

Extended Data Figure 3 | Biofinder image of cretaceous 95 Mya old shrimp fossil from Sannine formation, Hjoula, Lebanon illustrates that a combination of organics fluorescence signal along with morphology can help with the distinction of biotic vs. abiotic organic residue. In this image detection of the legs of shrimp in the fluorescence image is helpful in claiming this to be a biotic residue.
